# CXCL5-mediated recruitment of neutrophils into the peritoneal cavity of *Gdf15*-deficient mice protects against abdominal sepsis

**DOI:** 10.1073/pnas.1918508117

**Published:** 2020-05-18

**Authors:** Isa Santos, Henrique G. Colaço, Ana Neves-Costa, Elsa Seixas, Tiago R. Velho, Dora Pedroso, André Barros, Rui Martins, Nuno Carvalho, Didier Payen, Sebastian Weis, Hyon-Seung Yi, Minho Shong, Luís F. Moita

**Affiliations:** ^a^Innate Immunity and Inflammation Laboratory, Instituto Gulbenkian de Ciência, 2780-156 Oeiras, Portugal;; ^b^Serviço de Cirurgia Geral, Hospital de São Bernardo–Centro Hospitalar de Setúbal EPE, 2910-446 Setúbal, Portugal;; ^c^Serviço de Cirurgia Geral, Hospital Garcia de Orta, 2801-951 Almada, Portugal;; ^d^Faculdade de Medicina, Universidade de Lisboa, 1649-004 Lisboa, Portugal;; ^e^INSERM, UMR 1160, Universite Paris 7 Denis Diderot, Universite-Sorbonne Cité, 75013 Paris, France;; ^f^Institute for Infectious Disease and Infection Control, Jena University Hospital, 07747 Jena, Germany;; ^g^Department of Anesthesiology and Intensive Care Medicine, Jena University Hospital, 07747 Jena, Germany;; ^h^Center for Sepsis Control and Care, Jena University Hospital, 07747 Jena, Germany;; ^i^Research Center for Endocrine and Metabolic Diseases, Chungnam National University School of Medicine, 35015 Daejeon, Korea;; ^j^Instituto de Histologia e Biologia do Desenvolvimento, Faculdade de Medicina, Universidade de Lisboa, 1649-004 Lisboa, Portugal

**Keywords:** sepsis, GDF15, neutrophils, CXCL5

## Abstract

Sepsis is a life-threatening organ dysfunction condition caused by a dysregulated host response to an infection. Here we report that the circulating levels of growth and differentiation factor-15 (GDF15) are strongly increased in septic shock patients and correlate with mortality. In mice, we find that peptidoglycan is a potent ligand that signals through the TLR2-Myd88 axis for the secretion of GDF15, and that *Gdf15*-deficient mice are protected against abdominal sepsis due to increased chemokine CXC ligand 5 (CXCL5)-mediated recruitment of neutrophils into the peritoneum, leading to better local bacterial control. Our results identify GDF15 as a potential target to improve sepsis treatment. Its inhibition should increase neutrophil recruitment to the site of infection and consequently lead to better pathogen control and clearance.

## Significance

Sepsis remains a leading cause of death. New insights into its pathophysiology are likely to be key to the development of effective therapeutic strategies against sepsis. Given the role of GDF15 in metabolism regulation and in cachexia during late stages of cancer, features that also occur in sepsis, elucidation of the possible mechanistic role of GDF15 in sepsis is of great importance. We find that septic patients have very high levels of GDF15 in the peripheral blood, which correlate with clinical outcomes. Using *Gdf15*-deficient mice, we show that GDF15 plays a causal role in sepsis by delaying the local control of infection. These findings suggest GDF15 as a potential therapeutic target in sepsis secondary to a bacterial infection.

Sepsis is a complex disorder caused by a nonadaptive host response to an infection, leading to acute organ dysfunction and consequent high risk of death (1). It is the leading cause of death in intensive care units and the third-leading cause of overall hospital mortality (2). The pathophysiology and molecular bases of sepsis remain poorly understood. The urgently needed novel therapies for sepsis can only be inspired by new insights into the molecular bases of multiorgan failure and endogenous tissue protective mechanisms.

Organism survival of severe infection requires synergy between two evolutionarily conserved defense strategies that can limit host disease severity. Resistance requires inflammation and relies on reducing pathogen load, while disease tolerance provides host tissue damage control and limits disease severity irrespective of pathogen load (3). Resistance mechanisms have been extensively explored in the context of a response against infection and have been assumed to represent the extent of an immune response, while disease tolerance has received little attention until recently, and its cellular and molecular mechanisms are for the most part not understood (3). Recent efforts to mechanistically characterize the role of disease tolerance in survival of infection have uncovered important roles for metabolic regulation (4). Interestingly, metabolic dysfunction in sepsis has been extensively documented and might constitute a mechanistic basis for the multiorgan failure in sepsis (5).

Growth and Differentiation Factor 15 (GDF15) is known to regulate metabolism and energy homeostasis (6) and thus is a good candidate to play a role in sepsis. The relevance of this hypothesis is underscored by the fact that anorexia and long-lasting loss of muscle mass are frequently observed in sepsis (7). These features also characterize late stages of cancer, where GDF15 has been shown to play a critical role (8).

GDF15, also known as NSAID-activated gene 1 (NAG-1) and macrophage inhibitory cytokine 1 (MIC-1), is a stress response cytokine and a distant member of the transforming growth factor beta (TGFβ) superfamily (6) with well-documented roles in obesity and regulation of energy homeostasis (9), in addition to several conditions with an important inflammatory component (10). Four independent laboratories have recently identified glial-derived neurotrophic factor (GDNF) receptor alpha-like (GFRAL) as a central receptor for GDF15, likely using the tyrosine kinase receptor Ret as a coreceptor (11–14). Signaling through GFRAL accounts for the central actions of GDF15, including suppression of food intake, but is unlikely to also explain the peripheral effects of GDF15 (15). GDF15 serum levels are known to be elevated in many disease processes that course with cellular stress, most prominently cancer and cardiovascular diseases (16). The common pathological features between cancer (where GDF15 has a demonstrated role) and sepsis led us to ask whether GDF15 level is regulated in sepsis and whether it plays a causative role in its pathophysiology.

## Results

### GDF15 Levels Are Increased in Septic Shock Patients and Correlate with Mortality.

**Fig. 1.**
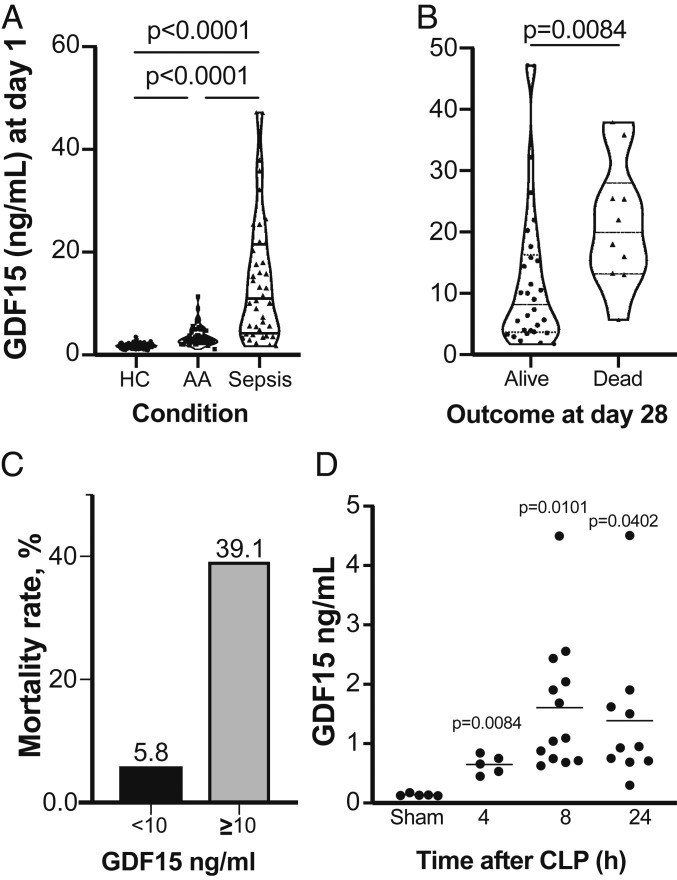
GDF15 is induced by infection in humans and mice. (*A*) Serum levels of GDF15 in the AA and severe sepsis groups compared with the HC group. (*B*) Serum levels of GDF15 in septic patients at day 1 of admission to the intensive care unit (ICU) comparing survival outcome at 28 d after admission to the ICU. (*C*) Comparison of mortality rates in patients with GDF15 serum levels <10 ng/mL and >10 ng/mL at day 1 of admission to the ICU. (*D*) GDF15 serum levels in mice subjected to CLP or sham surgery, quantified at the indicated time points. Each circle represents an individual mouse.

To begin addressing these questions, we measured GDF15 levels in 40 serum samples from septic patients and compared the results with those of 130 healthy controls (HC) and 33 patients with a histological diagnosis of acute appendicitis (AA; including 20 diagnosed with acute phlegmonous appendicitis and 13 with acute gangrenous appendicitis) before surgery. We found significant differences in GDF15 levels between AA and HC patients (*P* < 0.0001) and between AA and sepsis patients (*P* < 0.0001) (Fig. 1*A*). The septic patients had substantially higher values than the other groups (Fig. 1*A*), in good agreement with a recent report (17). We then examined survival of septic patients at 28 d after diagnosis and found that survivors had on average substantially lower GDF15 levels on the day of diagnosis (Fig. 1*B*). Importantly, patients with GDF15 <10 ng/mL at the day of diagnosis had a lower mortality rate at 28 d postdiagnosis compared with those with higher GDF15 levels (5.8% vs. 39.1%; Fig. 1*C*). None of the clinical or analytical severity parameters of AA patients correlated with GDF15 level. When subdivided based on mortality, sepsis patients did not differ in terms of age (W = 191; *P* = 0.2054) or sex (*P* = 1) (*SI Appendix*, Table S1). Compared with nonsurvivors, survivors had a lower Simplified Acute Physiology Score (SAPS) II (W = 244.5; *P* = 0.0033) and Acute Physiology and Chronic Health Evaluation (APACHE) II score (W = 108; *P* = 0.0174), but did not differ significantly on the Sequential Organ Failure Assessment (SOFA) score at day 1 (W = 188; *P* = 0.0702).

### GDF15 Induction Is Mediated by a TLR2-Myd88 Pathway in Mice.

**Fig. 2.**
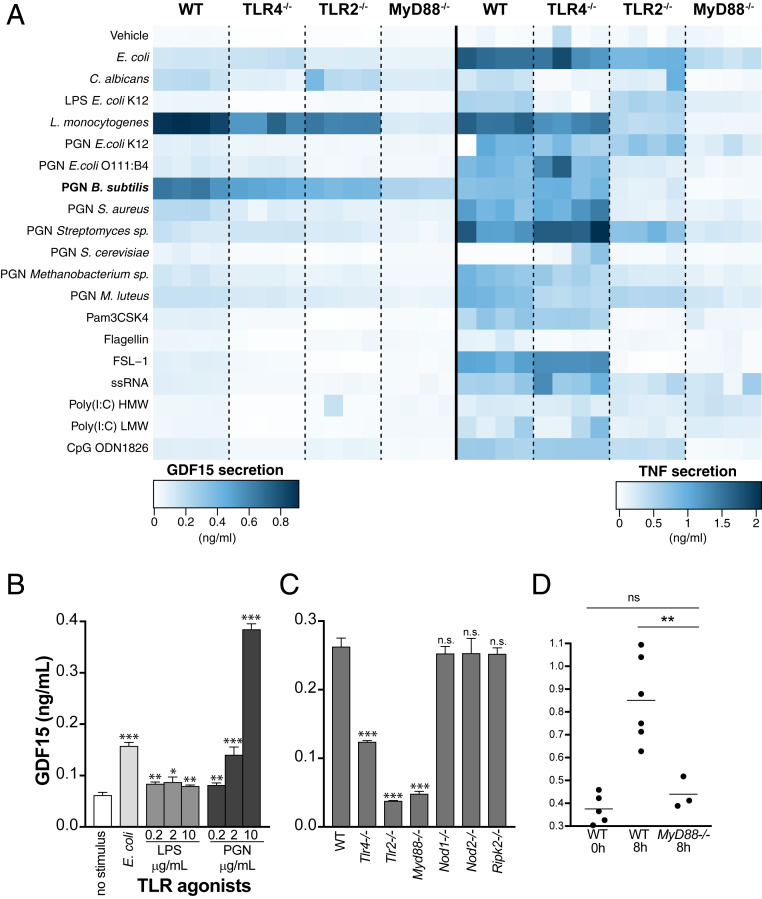
GDF15 is induced by PGN through a TLR2-Myd88 pathway in mice. Quantification by ELISA of GDF15 and TNF secretion induced by TLR agonists in BMDMs. (*A*) GDF15 and TNF levels in conditioned media following incubation of BMDMs from WT or knockout animals (*Tlr4*^*−/−*^, *Tlr2*^*−/−*^, and *Myd88*^*−/−*^) for 24 h without stimulus or with a series of TLR agonists. TLR agonists and concentrations: PFA-fixed *E. coli* at 20 bacteria per cell; PFA-fixed *C. albicans* at 20 yeast per cell; LPS from *E. coli* at 200 ng/mL; *Listeria monocytogenes* at 100 × 10^6^ bacteria/mL; PGN from a variety of microbial sources at 10, 5, and 2.5 μg/mL; Pam3CSK4 at 300 ng/mL; flagellin from *Salmonella typhimurium* at 1 μg/mL; FSL-1 (Pam2CGDPKHPKSF) at 100 ng/mL; ssRNA40 at 2.5 μg/mL; Poly(I:C) HMW at 10 μg/mL; Poly(I:C) LMW at 10 μg/mL; and CpG oligonucleotide at 1.5 μM. (*B*) GDF15 levels in conditioned media following incubation of WT BMDMs for 24 h without stimulus or with the following TLR agonists: PFA-fixed *E. coli* at a ratio of 20 bacteria per cell, LPS from *E. coli* at 0.2 to 10 μg/mL, and PGN from *B. subtilis* at 0.2 to 10 μg/mL. (*C*) GDF15 levels quantified as in *B*, following incubation of BMDMs from WT or knockout animals (*Myd88*^*−/−*^, *Tlr2*^*−/−*^, *Tlr4*^*−/−*^, *Nod1*^*−/−*^, *Nod2*^*−/−*^, and *Ripk2*^*−/−*^) for 16 h with PGN 2.5 μg/mL. Averages and SDs are shown for three replicate wells in 96-well plates; one representative experiment is shown, and at least three independent experiments were performed and quantified by ELISA. (*D*) GDF15 levels at 8 h after CLP in the peripheral blood of WT and *MyD88*^*−/−*^ mice. Each circle represents an individual mouse. n.s., not significant; *P < 0.05; **P < 0.01; ***P < 0.001.

To explore the role of GDF15 in sepsis, we first measured its levels at 4, 8, and 24 h in the peripheral blood of mice subjected to cecal ligation and puncture (CLP) and in mice subjected to sham operation alone (Fig. 1*D*). We found that similarly to TNF in serum after CLP (*SI Appendix*, Fig. S1*A*), GDF15 levels were significantly increased (Fig. 1*D*), in agreement with the findings in sepsis patients (Fig. 1*A*). We then used a panel of microbial pattern recognition agonists to challenge bone marrow-derived macrophages (BMDMs) from wild-type (WT) C56BL/6 mice and found that TLR2 agonists were the strongest inducers of GDF15 expression and secretion, in contrast to the broad pattern of TNF secretion measured in the same conditioned media (Fig. 2*A*). Based on these results, we chose peptidoglycan (PGN) from *Bacillus subtilis* for further experiments (Fig. 2*B*). In contrast with either *Escherichia coli* or increasing concentrations of lipopolysaccharide (LPS) that robustly induce the secretion of TNF (*SI Appendix*, Fig. S1*B*) but do not induce the secretion of GDF15, PGN from *B. subtilis* strongly induces the secretion of GDF15 in a concentration-dependent manner (Fig. 2*B*).

We then asked which pathway senses PGN leading to the secretion of GDF15. Compared with BMDM from WT C56BL/6, we found that the TLR2-MyD88 pathway is required for GDF15 secretion, while both NOD1 and NOD2 are dispensable (Fig. 2*C*). Depending on the dose and duration of challenge with PGN, TLR4-MyD88 is also capable of sensing PGN to induce GDF15 secretion (Fig. 2*C*).

To assess the significance of these findings in vivo, we measured the levels of GDF15 at 8 h after CLP in the peripheral blood WT and *MyD88*^*−/−*^ mice. We found a substantial and significant reduction in the circulating levels of GDF15 in *MyD88*^*−/−*^ mice in response to CLP compared with WT mice (Fig. 2*D*).

### *Gdf15*-Deficient Mice Are Protected Against CLP.

**Fig. 3.**
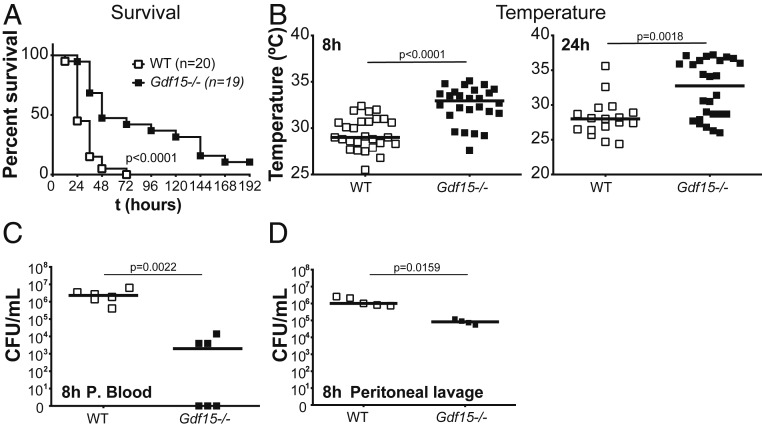
*Gdf15*^*−/−*^ mice are protected against CLP and have decreased CFUs in the peritoneum. (*A*) Survival to CLP comparing WT (*n* = 20) and *Gdf15*^*−/−*^ (*n* = 19) animals. (*B*) Rectal temperature in WT (*n* = 28) and *Gdf15*^*−/−*^ (*n* = 26) animals 8 and 24 h after CLP. (*C*) CFUs cultured from peripheral blood of WT (*n* = 6) and *Gdf15*^*−/−*^ (*n* = 6) animals at 8 h after CLP. (*D*) CFUs cultured from peritoneal lavage of WT (*n* = 5) and *Gdf15*^*−/−*^ (*n* = 4) mice at 8 h after CLP.

We then turned to the mechanistic role of GDF15 in sepsis, as the strongly increased secretion could mean either that this factor is involved in a compensatory response to a stress (infection) challenge or that it plays an active part in increasing the severity of infection. To distinguish between these two possibilities, we compared the survival rates of WT and *Gdf15*-deficient (*Gdf15*^*−/−*^) mice in response to a polymicrobial peritonitis using the CLP model of sepsis. In multiple and independent experiments, we consistently observed that *Gdf15*^*−/−*^ mice were protected against sepsis and survived for considerably longer periods (Fig. 3*A*). Sickness behavior is defined as a group of signs and symptoms, including anorexia, lethargy, fever, altered sleep patterns, lack of grooming, and social withdrawal, in response to an infection. In line with this, *Gdf15*^*−/−*^ mice showed reduced temperature loss compared with WT mice at both 8 h and 24 h after CLP (Fig. 3*B*). *Gdf15*^*−/−*^ mice had lower scores for sickness behavior, as detailed in *SI Appendix*, Fig. S2. All tested cytokines had lower levels in *Gdf15*^*−/−*^ mice (*SI Appendix*, Fig. S3), which reached statistical significance in the cases of IL-1β and IL-12 (*SI Appendix*, Fig. S3 *C* and *F*) suggesting better control of infection in *Gdf15*^*−/−*^ mice. We found no relevant differences in the levels of serologic markers of organ dysfunction or damage (including creatinine, LDH, CK, AST, and ALT) at 24 h, suggesting similar organ damage in the two strains (*SI Appendix*, Fig. S4 *A*–*E*). This result is in agreement with minor histological differences between the genotypes at 24 h after CLP (*SI Appendix*, Fig. S4*F*).

### *Gdf15*-Deficient Mice Show Better Control of the Local Infection.

We then measured the bacterial load in the peripheral blood of WT and *Gdf15*^*−/−*^ mice in independent experiments and found consistently a statistically significant lower bacterial burden in *Gdf15*^*−/−*^ mice at 8 h after CLP (Fig. 3*C*), suggesting that better control of the initial local infection in *Gdf15*^*−/−*^ mice. Taken together, the results for cytokines, serologic markers of organ lesion, histopathology, and bacterial burden suggest that *Gdf15*^*−/−*^ mice are more resistant to infection without affecting disease tolerance, as described above (3).

**Fig. 4.**
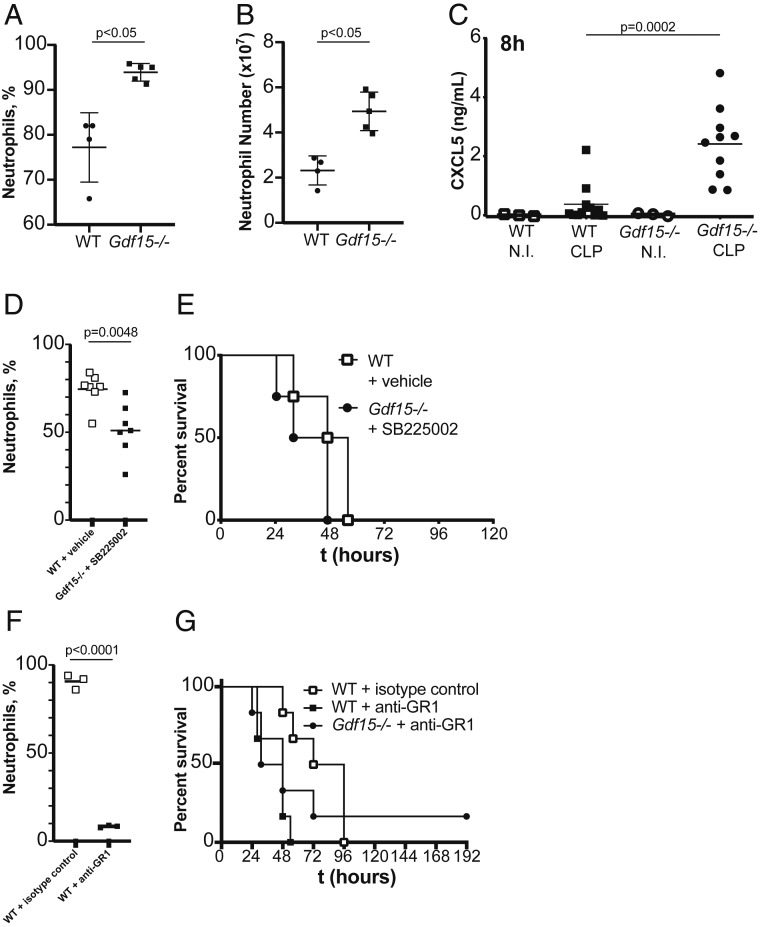
*Gdf15*^*−/−*^ mice demonstrate better control of local infection due to CXCL5-mediated neutrophil influx into the peritoneum. (*A* and *B*) Percentage (*A*) and total number of neutrophils (*B*) in the peritoneal lavage of WT (*n* = 4) and *Gdf15*^*−/−*^ (*n* = 5) mice at 8 h after CLP. (*C*) ELISA quantification of CXCL5 in the peritoneal lavage of WT and *Gdf15*^*−/−*^ mice at 8 h after CLP or control (noninfected; NI) . (*D*) Number of neutrophils in peritoneal lavage after CLP in WT vehicle-treated mice (WT; *n* = 7) and *Gdf15*^*−/−*^ mice treated with CXCR2 inhibitor SB225002 (*Gdf15*^*−/−*^; *n* = 6). (*E*) Survival to CLP in WT vehicle-treated mice (WT; *n* = 4) and *Gdf15*^*−/−*^ mice treated with CXCR2 inhibitor SB225002 (*Gdf15*^*−/−*^; *n* = 4). (*F*) Number of neutrophils in peritoneal lavage after CLP in WT isotype control-treated mice (*n* = 3) and WT mice treated with anti-GR1 antibody (*n* = 3). (*G*) Survival to CLP in WT isotype control-treated mice (*n* = 6) and WT anti-GR1–treated mice (*n* = 6) and *Gdf15*^*−/−*^ mice treated anti-GR1 antibody (*n* = 6).

To further investigate this possibility, we analyzed and compared the peritoneal lavage contents of WT and *Gdf15*^*−/−*^ mice after 8 h of CLP. We found bacterial levels on the peritoneal lavage to be on average 10-fold lower (*P* = 0.0159) in *Gdf15*^*−/−*^ mice (Fig. 3*D*), in good agreement with the substantially elevated relative (Fig. 4*A*; *P* < 0.05) and absolute (Fig. 4*B*; *P* < 0.05) numbers of neutrophils. The increased number of neutrophils, rather than their differential activity between genotypes, is likely responsible for a better local control of the initial infection, as we did not observe increased phagocytic activity.

### *Gdf15*-Deficient Mice Show Increased CXCL5-Mediated Neutrophil Recruitment.

To identify mechanistic bases for the different numbers of neutrophils at the site of infection, we measured the main chemokine factors for neutrophil attraction, including the CCR1 ligands CCL3 (*SI Appendix*, Fig. S5*A*), CCL4 (*SI Appendix*, Fig. S5*B*), and CCL5 (*SI Appendix*, Fig. S5*C*) and the CXCR2 ligands MIP-2/CXCL-2 (*SI Appendix*, Fig. S5*D*), KC/CXCL-1 (*SI Appendix*, Fig. S5*E*), and CXCL5 (Fig. 4*C*). GDF15 was increased systemically only in WT animals and was completely absent in knockout animals (*SI Appendix*, Fig. S5*F*). IL-6 and MCP-1, a chemokine for macrophages, were very similar, suggesting that the CLP procedure was done with similar severity in both genotypes (*SI Appendix*, Fig. S5 *G* and *H*). Only LIX/CXCL5 showed a strong, consistent, and significant increase in *Gdf15*^*−/−*^ mice (*P* = 0.0002; Fig. 4*C*); all the other chemokines were either very similar or even marginally decreased (*SI Appendix*, Fig. S5).

To test whether increased signaling through the CXCL5-CXCR2 axis is directedly responsible for the *Gdf15*^*−/−*^ protective phenotype, we used a CXCR2-selective antagonist, SB225002, and compared the percent of neutrophils in the peritoneal cavity (Fig. 4*D*) and survival (Fig. 4*E*) of WT and *Gdf15*^*−/−*^ mice after CLP and treatment with this CXCR2 antagonist. We observed that the CXCR2-selective antagonist inhibited the excess neutrophil recruitment observed in *Gdf15*^*−/−*^ (Fig. 4*D*), and that in these conditions the survival advantage of *Gdf15*^*−/−*^ mice to CLP was lost (Fig. 4*E*), suggesting that increased local levels of CXCL5 in *Gdf15*^*−/−*^ mice is a necessary component of their increased survival of a peritoneal polymicrobial infection. This conclusion is also supported by neutrophil depletion experiments. When CLP-induced neutrophil recruitment was almost completely eliminated by an anti–GR1-depleting antibody (Fig. 4*F*), mice died substantially faster than WT mice that had been treated with an isotype control antibody and, importantly, no differences in survival between genotypes were observed, supporting the hypothesis that increased neutrophil recruitment to the site of infection in *Gdf15*^*−/−*^ mice accounts for their survival advantage in CLP.

## Discussion

In the present study, we found that *Gdf15*^*−/−*^ mice are protected against the CLP model of sepsis because they are better able to control the initial infection by locally secreting higher levels of the CXCL5 chemokine, known to be involved in neutrophil recruitment through the activation of the CXCR2 receptor (18). Our findings and conclusions are consistent with previous observations in a mouse model of myocardial infarction where *Gdf15*^*−/−*^ mice recruited more neutrophils to the injury site (19). In this model, mice had a worse outcome as the excess number of neutrophils exacerbated the myocardial lesion (19). Excess recruitment of neutrophils has also been observed in *Gdf15*^*−/−*^ mice in a model of CCl_4_-induced liver fibrogenesis (20), which contributed to lesion exacerbation. In contrast, in the present CLP model, the increased number of neutrophils led to better early control of the local infection. Taken together, our findings and previously published observations converge on the modulation of neutrophil recruitment, supporting a role for GDF15 in the regulation of tissue injury and indicating that its effects are likely to be highly contextual.

We still do not understand how GDF15 negatively regulates CXCL5-mediated neutrophil recruitment to the site of infection, but previous reports have shown that GDF15 regulates neutrophil arrest and platelet aggregation under flow conditions by modulating the affinity of integrins (19, 21, 22). At this point, it is unclear how effects on integrins relate to CXCL5 modulation, but data suggest that the effects of GDF15 on CXCL5 levels are likely indirect and non–cell-autonomous.

The significance of our findings is highlighted by the knowledge that neutrophils are critical innate immune cells that provide the first line of host defense against sepsis through their ability to rapidly migrate to the site of infection; in addition, many animal and clinical sepsis studies have indicated that neutrophils display markedly impaired recruitment to infectious sites and fail to clear bacteria, resulting in excessive inflammation and increased mortality (23), findings that lacked a mechanistic basis before the present study. Our results raise the possibility that GDF15 may play a key detrimental effect in sepsis by inhibiting neutrophil recruitment to the site of infection, which constitutes an important immunosuppression component that characterizes late stages of sepsis and is responsible for the inability to control and clear infection (24). Strategies to reverse the defects in migration of neutrophils have been proposed as promising therapies for sepsis (25). Our work indicates GDF15 as a possible target for this goal. In the present study, we used a model of peritoneal sepsis; further work is needed to test the significance of GDF15 regulation on CXCL5-mediated neutrophil recruitments for other origins of infection, including lung bacteria pneumonia. Our study also not did not address the mechanistic role of GDF15 in sepsis caused by other groups of pathogens, including viruses and fungi, which are increasingly frequent causes of this condition.

Another important implication of our work relates to the identification of CXCL5 as the causal mediator responsible for increased survival of *Gdf15*^*−/−*^ mice, suggesting that the expression of this chemokine is suppressed by GDF15. CXCL5 has been directly implicated in obesity and insulin resistance (26). This fact is particularly interesting in the context of our findings, possibly meaning that the obesity phenotype of *Gdf15*^*−/−*^ mice does not depend exclusively on the absence of signaling through the central GFRAL central receptor, but also depends on a peripheral inflammatory component mediated by CXCL5. In fact, while *Gdf15*^*−/−*^ animals have increased body weight when fed a standard chow diet, mice lacking GFRAL do not differ in body weight or composition compared with WT animals fed a standard diet (12–14).

The observations and the implications of our findings are in clear contrast with the recent work of Luan et al. (27), who found that GDF15 enhances disease tolerance through the modulation of liver lipid metabolism and triglyceride availability during infection (28). While it is possible that the opposing results may reflect nonredundant effects of GDF15 revealed by different experimental settings, indicating that GDF15 can be cardioprotective (27) and also negatively regulate neutrophil recruitment (19, 20), they perhaps more likely can be explained by the use of a blocking antibody (27) or *Gdf15-*deficient mice (as seen in the present study). Whatever the true biological role of GDF15 in infection, it is clear that its conclusive elucidation is critical as the modulation of GDF15 is rapidly being tested in clinical trials for different conditions (28).

## Methods

### Mice.

*Gdf15*^−/−^ mice were derived from the inbred C57BL/6 strain and were originally provided by S. Lee, Johns Hopkins University School of Medicine. *Gdf15*^*−/−*^, *Myd88*^*−/−*^, *Tlr2*^*−/−*^, *Tlr4*^*−/−*^, and C57BL/6J control mice were bred and maintained under specific-pathogen free conditions at the Instituto Gulbenkian de Ciência on a 12-h light/12-h dark cycle, humidity 50 to 60%, ambient temperature 22 ± 2 °C, and food and water ad libitum.

### CLP.

Bedding of age-matched C57BL/6J and *Gdf15*^*−/−*^ mice was mixed 2 wk before the start of the experiments to mitigate differences in microbiome composition. Polymicrobial sepsis was induced in mice by CLP as described previously (29).

### Neutrophil Depletion In Vivo.

For the depletion of neutrophils in vivo, mice were injected i.p. with 26 mg/g of anti-mouse Ly6G mAb or isotype control. The efficacy of the neutrophil depletion was confirmed by flow cytometry.

### Sickness Behavior Assay.

The sickness behavior assay used in this report has been described previously (30). In brief, mice were observed by two researchers and rated for the presence (1) or absence (0) of each of the following symptoms: piloerection, ptosis, lethargy, and huddling. The score was recorded as the sum of the four parameters.

### Colony Forming Units Assay.

Colony-forming units (CFU) were determined in blood and peritoneal lavage by serially diluting in sterile PBS and plating in trypticase soy agar plates with 5% sheep’s blood. Four dilutions were plated per condition. CFU were counted after incubating plates at 37 °C for 16 h.

### RT-qPCR Analysis.

Frozen mouse tissues were lysed in TRIzol (Invitrogen) using a TissueLyser II (QIAGEN), and RNA was purified with the RNeasy Mini Kit (QIAGEN). cDNA was synthesized with SuperScript II reverse transcriptase (Invitrogen) and quantified in the QuantStudio 7 system (Applied Biosystems). Relative *Gdf15* levels were normalized to GAPDH levels. The sequences of the primers used for *Gdf15* amplification were CTG​GCA​ATG​CCT​GAA​CAA​CG (forward) and GGT​CGG​GAC​TTG​GTT​CTG​AG (reverse).

### BMDM Treatments.

BMDMs were plated in supplemented RPMI 1640 medium (Gibco) and treated 16 h later for the indicated times. The agonists used were from the mouse TLR1-9 Agonist Kit (Invivogen; tlrl-kit1mw), and PGN was obtained from a variety of sources. All PGN reagents were obtained from Sigma-Aldrich and from a series of microbial origins: *Bacillus subtilis*, (catalog no. 69554), *Staphylococcus aureus* (77140), *Micrococcus luteus* (53243), *Streptomyces* sp. (79682), *Methanobacterium* sp. (78721), and *Saccharomyces cerevisiae* (72789). PFA-fixed *E. coli* was incubated with the BMDMs at a ratio of 20 bacteria per cell for the indicated times.

### Biochemical Assays in Serum, Peritoneal Lavage, and Supernatant from BMDMs.

GDF15 levels were quantified in the serum of patients using the GDF15 Human ELISA Kit (R&D Systems; catalog no. DY957) according to the manufacturer’s protocol. Whole mouse blood (obtained by cardiac puncture) was centrifuged at 1,600 × *g* for 5 min at 4 °C, and serum was collected and stored at −80 °C before analysis. Peritoneal lavage was centrifuged at 300 × *g* for 5 min at 4 °C, and the supernatant was collected and stored at −80 °C before analysis. For neutrophil identification, we used the following antibodies: CD11b, Ly6G, Ly6C, GR1, and anti-neutrophil (7/4). We calculated the absolute number of cells by flow cytometry using Perfect-Count Microspheres (Chromocyte). The absolute number of the cell population of interest was determined by dividing the number of cells of interest acquired by the number of Perfect-Count Microspheres acquired and multiplying this result by the microsphere concentration. Cytokine and chemokine levels were determined using the following ELISA kits, according to the manufacturer’s instructions: mouse TNF-α (430902; BioLegend), IL-1β (432601; BioLegend), IL-6 (431302; BioLegend), IL-10 (431411; BioLegend), IL-12/IL-23 (p40) (431602; BioLegend), CKCL-5/LIX (DY443; R&D Systems), MCP-1 (432701; BioLegend), CCL3/MIP-1α (DY450; R&D Systems), mouse CCL4/MIP-1β (DY451; R&D Systems), mouse CCL5/RANTES (DY478; R&D Systems), CLCX-1/KC (DY453; R&D Systems), CXCL-2/MIP-2 (DY452; R&D Systems), and mouse GDF-15 (DY6385; R&D Systems). Serologic markers of organ damage/dysfunction were determined using the following colorimetric kits, according to the manufacturer’s instructions: QuantiChrom creatinine (DICT; Bioassay Systems), QuantiChrom lactate dehydrogenase (D2DH; Bioassay Systems), EnzyChrom creatine kinase (ECPK; Bioassay Systems), EnzyChrom alanine transaminase (EALT; Bioassay Systems), and EnzyChrom aspartate transaminase (EALT; Bioassay Systems). All absorbance readings were performed in 96-well plates using an Infinite M200 plate reader (Tecan).

### Flow Cytometry.

Cells were incubated with different antibodies diluted in PBS containing 2% FBS and 0.01% NaN_3_. To prevent nonspecific binding, cells were incubated at 4 °C for 20 min with CD16/CD32 to block Fc receptors. Then cells were incubated for 30 min at 4 °C with the following fluorescent-labeled antibodies: anti–CD49b-PE, anti–CD3-PE, anti–B220-PE, anti–-Ly6C-APC/Cy7, anti–Ly6G-APC, anti–F4/80-FITC, anti–CD11b-PerCp, anti–CD11b-BV785, anti–F4/80-A647, anti–GR1-PerCp/Cy5.5, anti-neutrophil (7/4)-FITC, and Sytox blue. All antibodies were obtained from BioLegend except anti-neutrophil (7/4) from Abcam and B220, CD49b, and GR1 from BD Biosciences. Flow cytometry data were acquired with an LSR Fortessa X-20 (BD Biosciences) and analyzed using the FlowJo software package (Tree Star).

### Histopathology.

Mouse liver, lung, and kidney were collected at 24 h after CLP and immediately fixed in 10% buffered formalin. Samples were then embedded in paraffin, sectioned (3 μm), and stained with hematoxylin and eosin according to standard procedures. Histopathology analysis was performed by a trained pathologist blinded to the mice strain and infection challenge at the Instituto Gulbenkian de Ciência Histopathology Unit. Tissues were scored for damage, namely necrosis and leukocyte infiltration.

### Statistical Analysis.

The Mann–Whitney *U* test and Mantel–Cox test (for survival curves) were used for statistical analyses, performed with GraphPad Prism 6.0. A *P* value <0.05 was considered statistically significant.

### Study Approval.

Human sepsis samples were included under a project approved by the French Ethical committee (“‘Comité de Protection des Personnes,” Ile de France IV; 2010-A0004039). Samples from HCs and patients with AA were included under a research project approved by the Ethics Committee of Garcia de Orta Hospital, Portugal (reference 05/2015). Written informed consent was obtained from each enrolled patient or a family member. This work was carried out in accordance with the Code of Ethics of the World Medical Association (Declaration of Helsinki). All animal studies were performed in accordance with Portuguese regulations and approved by the Instituto Gulbenkian de Ciência Ethics Committee and Direção Geral de Alimentação e Veterinária (reference A002.2015).

### Data Availability.

The raw data used to support the findings of this study are restricted by the Ethics Committees of Hospital Garcia de Orta and “Comité de Protection des Personnes” Ile de France IV to protect patient privacy. All data are available from the lead author on reasonable request (lmoita@igc.gulbenkian.pt). Requests for patient data will only be considered for researchers who meet the criteria for access to confidential data.

## Supplementary Material

Supplementary File
