# Supplementary material for: CXCL5-mediated recruitment of neutrophils into the peritoneal cavity of Gdf15-deficient mice protects against abdominal sepsis
Source: Proc Natl Acad Sci U S A. 2020 May 18;117(22):12281–7. doi: 10.1073/pnas.1918508117 (PMC7275717; doi:10.1073/pnas.1918508117)
Supplement: Supplementary File [file pnas.1918508117.sapp.pdf]

SI Appendix for:

CXCL5-mediated recruitment of neutrophils into the peritoneal cavity of *Gdf15*-deficient mice protects against abdominal sepsis

Isa Santos[1,2,*], Henrique G. Colaço[1,*], Ana Neves-Costa[1,*], Elsa Seixas[1], Tiago R. Velho[1], Dora Pedroso[1], André Barros[1], Rui Martins[1], Nuno Carvalho[3,4], Didier Payen[5], Sebastian Weis[6,7,8], Hyon-Seung Yi[9], Minho Shong[9] and Luís Ferreira Moita[1,10,11].

[1]Instituto Gulbenkian de Ciência, Rua da Quinta Grande 6, 2780-156 Oeiras, Portugal
[2]Serviço de Cirurgia Geral, Hospital de São Bernardo - Centro Hospitalar de Setúbal, EPE, Portugal
[3]Serviço de Cirurgia Geral, Hospital Garcia de Orta, Almada, Portugal
[4]Faculdade de Medicina, Universidade de Lisboa, Portugal
[5]INSERM UMR 1160 University Paris 7 Denis Diderot, Paris-Sorbonne Cité, France
[6]Institute for Infectious Disease and Infection Control, Jena University Hospital, Germany
[7]Department of Anesthesiology and Intensive Care Medicine, Jena University Hospital, Germany
[8]Center for Sepsis Control and Care, Jena University Hospital, 07747 Jena, Germany
[9]Research Center for Endocrine and Metabolic Diseases, Chungnam National University School of Medicine, Daejeon 35015, Korea
[10]Instituto de Histologia e Biologia do Desenvolvimento, Faculdade de Medicina, Universidade de Lisboa, Portugal
[11]Corresponding author: Luis F. Moita, lmoita@igc.gulbenkian.pt
[*]These authors contributed equally to this work

www.pnas.org/cgi/doi/10.1073/pnas.1918508117

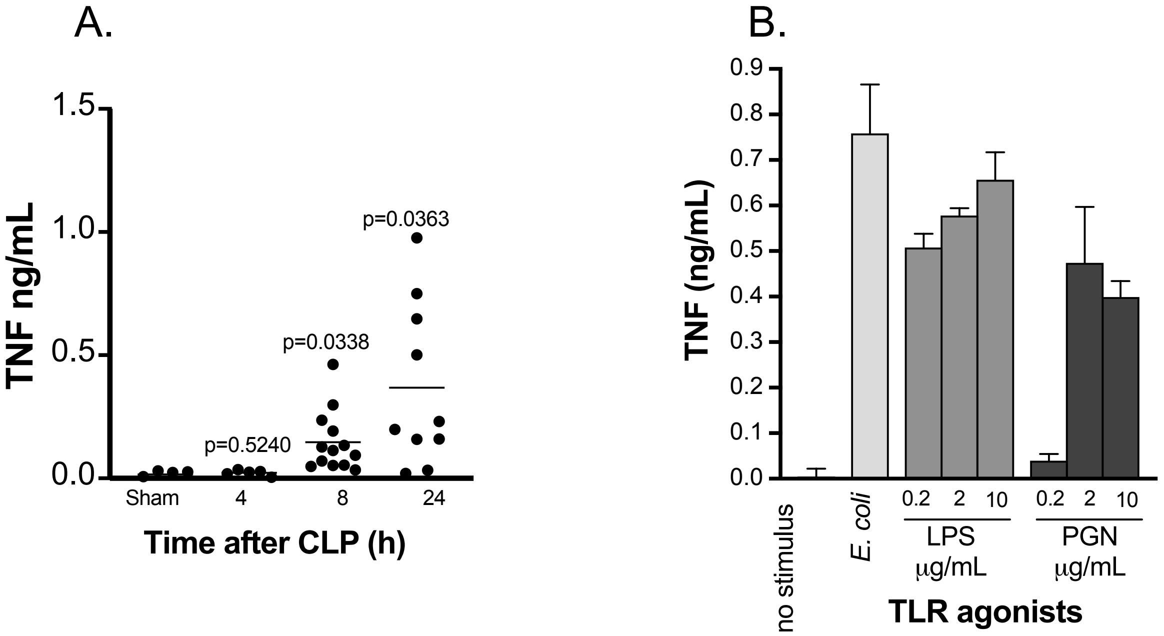

SI Appendix Fig. S1. TLR agonists induce GDF15.
(A) TNF serum levels in mice subjected to CLP or sham surgery, quantified at the indicated time points. Each individual is represented by a circle. (B) Quantification of TNF levels in the same conditions as in Fig. 2B.

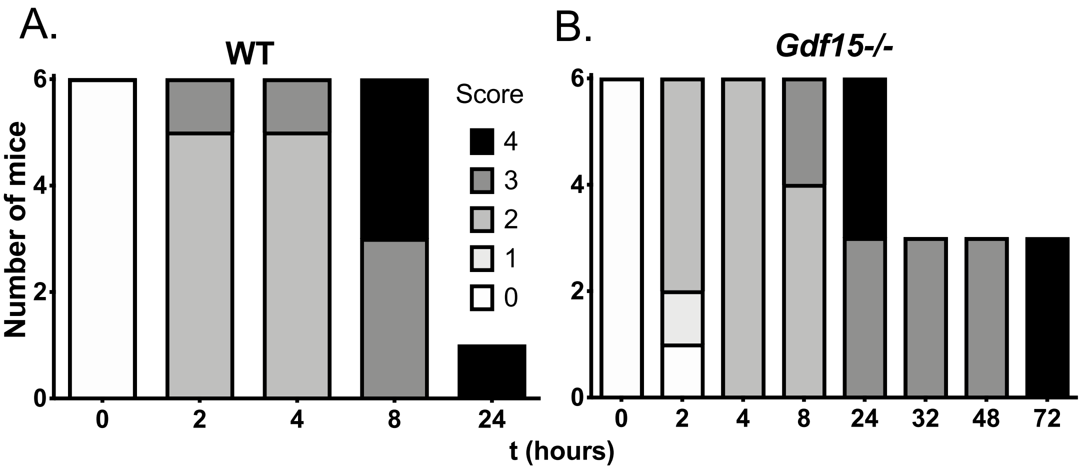

SI Appendix Fig. S2. GDF15 modulates sickness behavior.
A five-point scale to evaluate the development of sickness behaviors in WT (A) and Gdf15-/- mice (B). At specific time points following CLP, animals were examined by two observers who independently scored the presence of specific signs of sickness. In each animal, the following four signs were evaluated: (1) Piloerection, (2) Ptosis, (3) Lethargy and (4) Huddling. All animals were observed inside their cages. Each parameter was scored as presence (1) or absence (0). Graphs represent thedistribution of sickness behavior presented at each timepoint. “0” = no sickness behavior; “4” = all four signs of sickness behavior.

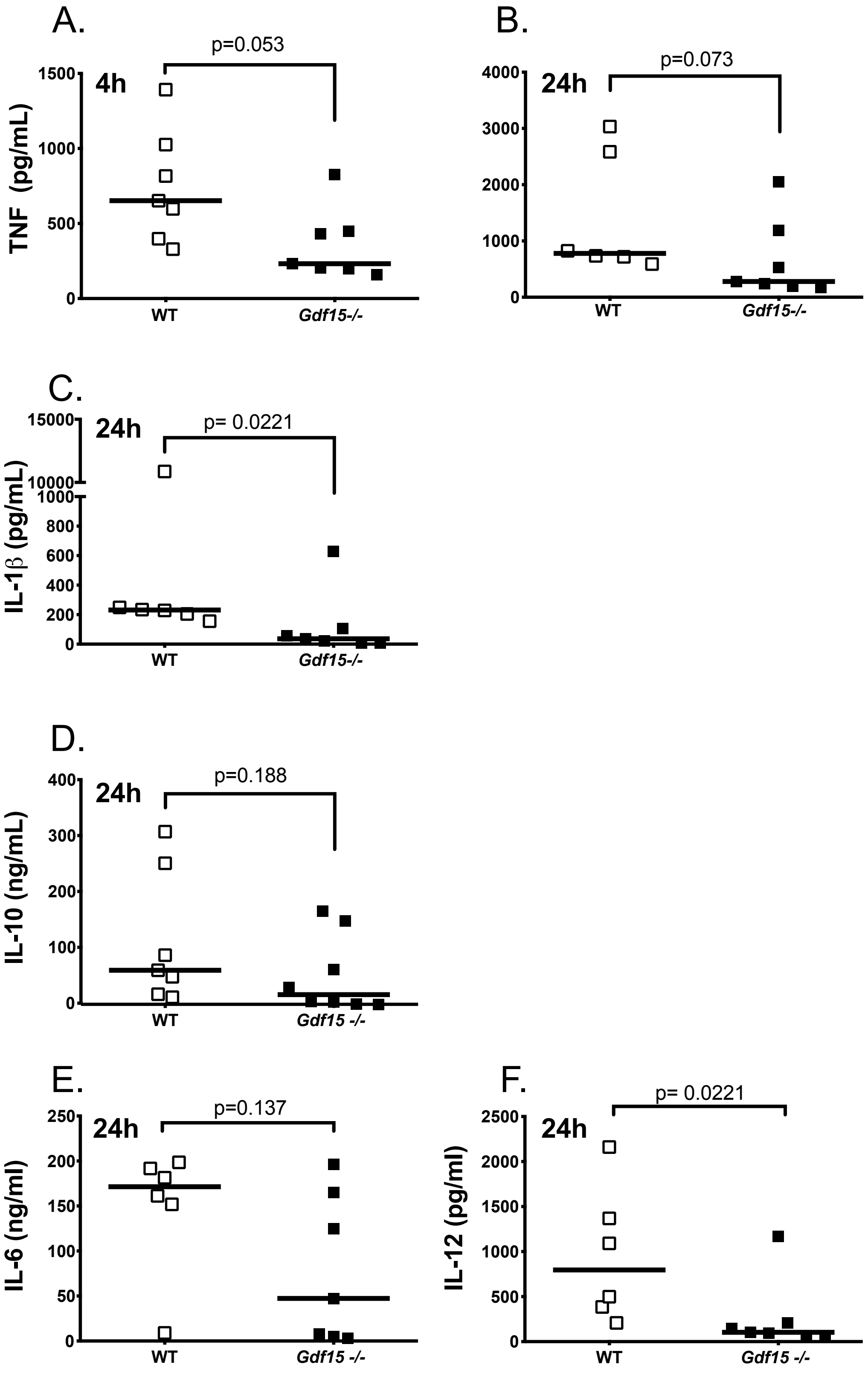

SI Appendix Fig. S3. Cytokine quantification in peripheral blood of animals after CLP. (A) Quantification by ELISA of TNF at 4 h, (B) TNF at 24 h, (C) IL-1β at 24 h, (D) IL-10 at 24 h, (E) IL-6 at 24 h and (F) IL-12 at 24 h after CLP in WT or Gdf15-/- mice.

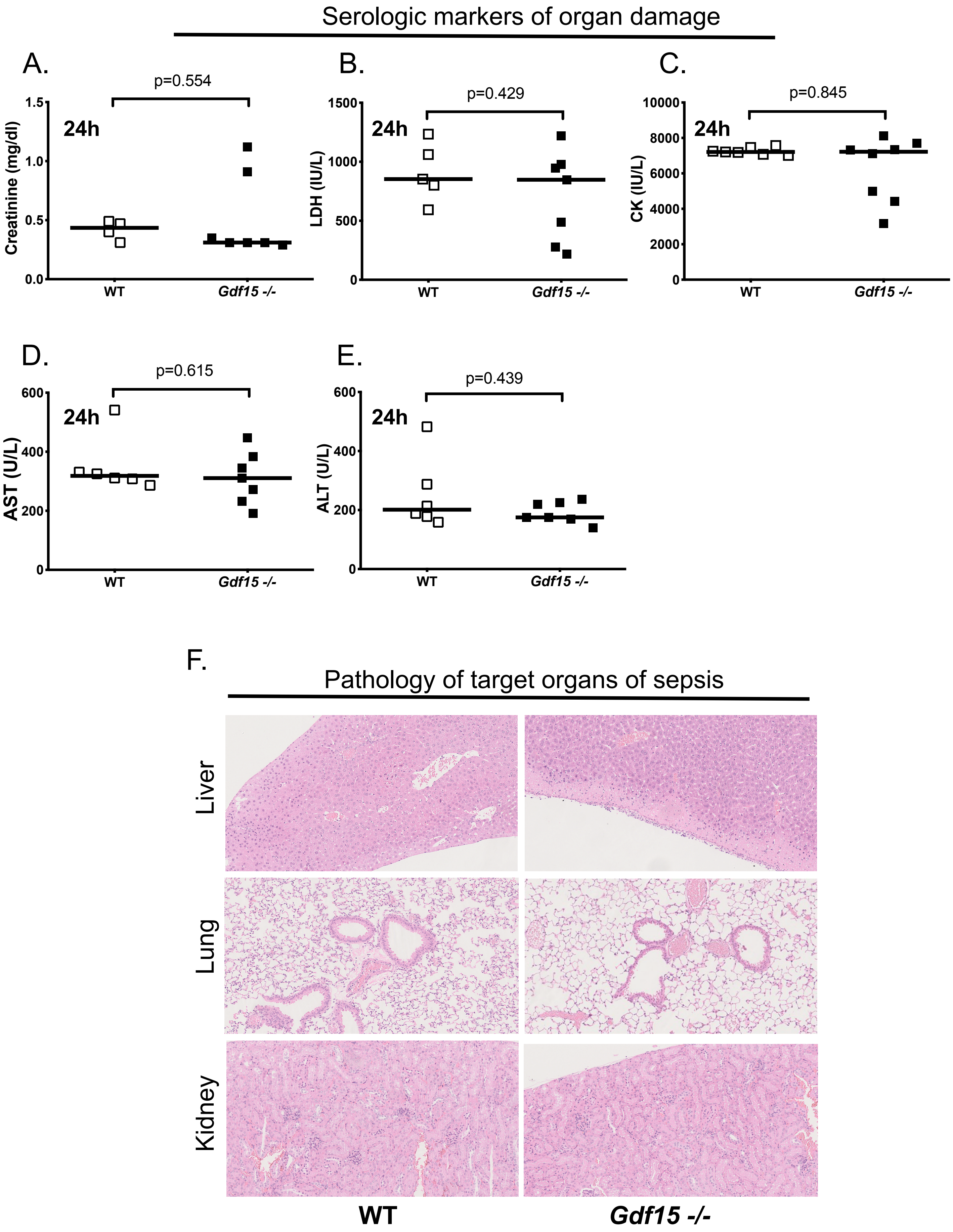

SI Appendix Fig. S4. WT and Gdf15-/- mice have similar degrees of tissue damage after CLP. (A), Colorimetric quantification of serum levels of creatinine, (B) LDH, (C) CK, (D) AST and (E) ALT 24 h after CLP in WT and Gdf15-/- mice. (F) Histology analysis of HE stains of liver, lung and kidney from WT and Gdf15-/- mice 24 h after CLP.

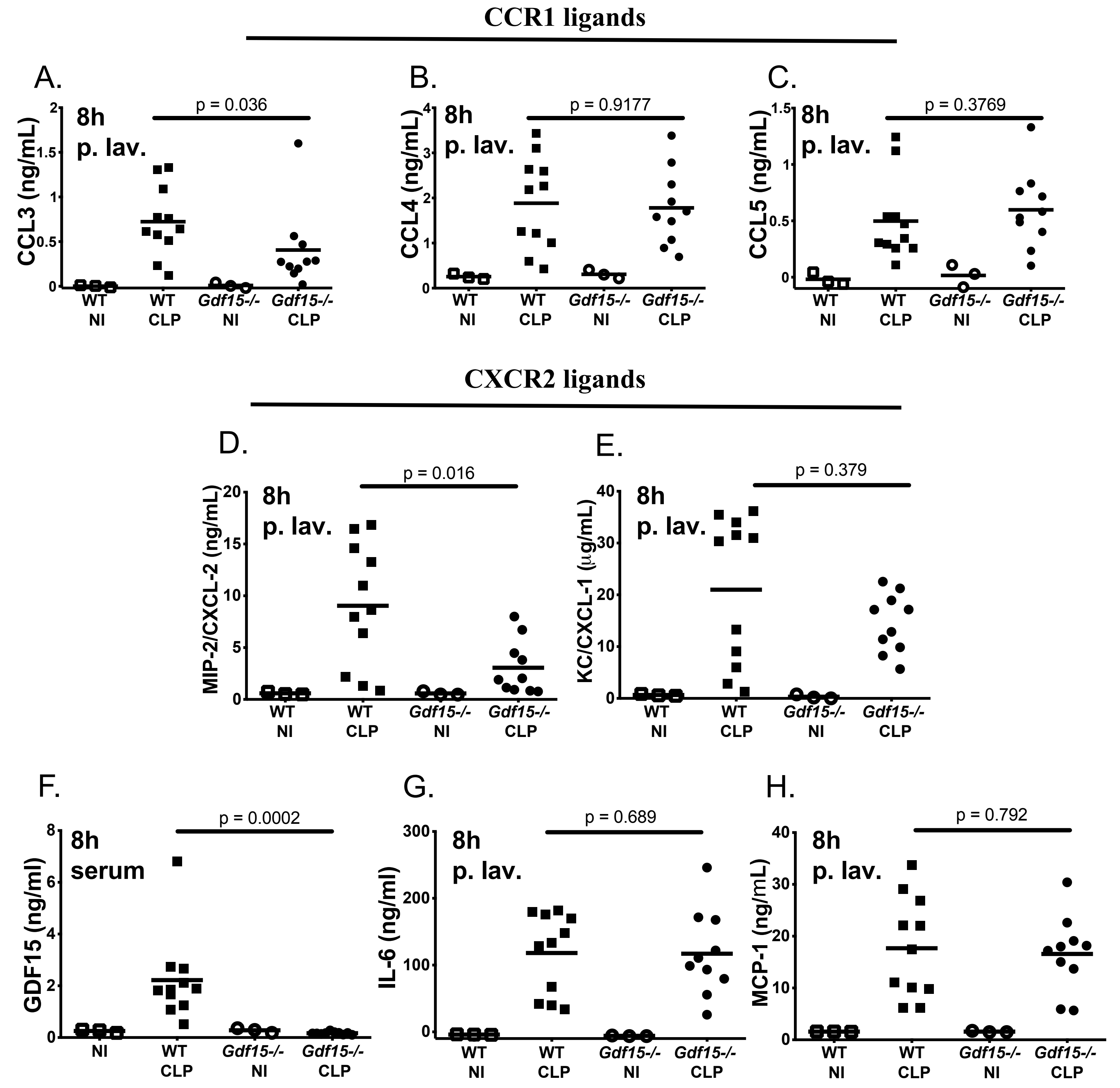

SI Appendix Fig. S5. Cytokine and chemokine quantification in the peritoneal lavage and peripheral blood from mice after CLP. Quantification by ELISA of the CCR1 ligands (A) CCL3, (B) CCL4, (C) CCL5 and the CXCR2 ligands (D) MIP-2/CXCL-2 and (E) KC/CXCL-1 in the peritoneal lavage of WT and Gdf15-/- mice 8 h after CLP. Quantification by ELISA of (F) GDF15, (G) IL-6 and (H) MCP-1 from WT and Gdf15-/- mice 8 h after CLP.

Supplementary Table I
Sepsis cohort characterization. Separation based on mortality at day 28.

**Variable**	**Non-survivors** (n=10)	**Survivors** (n=30)	*Statistical Testing*
Age	75.8 ± 13.39; 77 (72; 85.5)	65.73 ± 20.79; 71 (58.5; 80)	W = 191<br>p = 0.2054
Gender	4 Male : 6 Female	14 Male : 16 Female	Fisher Exact Test;<br>p=1
SAPS II	64.40 ± 11.18; 65.5 (60.25; 70.50)	50.6 ± 13.15; 53 (43.50; 58.50)	W = 244.5<br>p = 0.003289
APACHE II	31.71 ± 9.11 (3 NA); 34 (28; 35.50)	23.79 ± 4.84 (11 NA); 25 (21.5; 27)	W = 108<br>p = 0.01741
SOFA_D1	10.42 ± 3.53; 11.5 (8.25; 12.75)	8.33 ± 2.72 (3 NA); 8 (7; 10.5)	W = 188<br>p = 0.07023
